# Efficient removal of pharmaceutical contaminants from water and wastewater using immobilized laccase on activated carbon derived from pomegranate peels

**DOI:** 10.1038/s41598-023-38821-3

**Published:** 2023-07-24

**Authors:** Osamah J. Al-sareji, Mónika Meiczinger, Raed A. Al-Juboori, Ruqayah Ali Grmasha, Manolia Andredaki, Viola Somogyi, Ibijoke A. Idowu, Csilla Stenger-Kovács, Miklós Jakab, Edina Lengyel, Khalid S. Hashim

**Affiliations:** 1 Sustainability Solutions Research Lab, Faculty of Engineering, University of Pannonia, Egyetem str. 10, Veszprém, 8200 Hungary; 2 Environmental Research and Studies Center, University of Babylon, Al-Hillah, Babylon Iraq; 3 NYUAD Water Research Center, New York University-Abu Dhabi Campus, P.O. Box 129188, Abu Dhabi, United Arab Emirates; 4 Water and Environmental Engineering Research Group, Department of Built Environment, Aalto University, Aalto, P.O. Box 15200, 00076 Espoo, Finland; 5 Research Group of Limnology, Faculty of Engineering, Center for Natural Science, University of Pannonia, Egyetem u. 10, Veszprém, 8200 Hungary; 6 School of Civil Engineering and Built Environment, Liverpool John Moores University, Liverpool, UK; 7 ELKH-PE Limnoecology Research Group, Egyetem utca 10, Veszprém, 8200 Hungary; 8 Department of Materials Sciences and Engineering, Research Centre of Engineering Sciences, University of Pannonia, P.O. Box 158, Veszprém, 8201 Hungary; 9 Department of Environmental Engineering, College of Engineering, University of Babylon, Al-Hillah, Babylon, Iraq

**Keywords:** Pollution remediation, Environmental sciences

## Abstract

In this study, pomegranate peels (PPs) as an abundant fruit processing waste was used to produce cost-effective, eco-friendly, and high-quality activated carbon. The produced carbon (fossil free activated carbon) was used for immobilizing laccase to remove a range of emerging pollutants namely diclofenac, amoxicillin, carbamazepine, and ciprofloxacin from water and wastewater. The loaded activated carbon by laccase (LMPPs) and the unloaded one (MPPs) were characterized using advanced surface chemistry analysis techniques. MPPs was found to have a porous structure with a large surface area and an abundance of acidic functional groups. Laccase immobilization reduced surface area but added active degradation sites. The optimal immobilization parameters were determined as pH 4, 35 °C, and a laccase concentration of 2.5 mg/mL resulting in a 69.8% immobilization yield. The adsorption of the emerging pollutant onto MPPs is best characterized as a spontaneous endothermic process that adheres to the Langmuir isotherm and first-order kinetics. Using synergistic adsorption and enzymatic degradation, the target pollutants (50 mg/L) were eliminated in 2 h. In both water types, LMPPs outperformed MPPs. This study shows that pomegranate peels can effectively be harnessed as an enzyme carrier and adsorbent for the removal of emerging pollutants even from a complex sample matrix. The removal of contaminants from wastewater lasted five cycles, whereas it continued up to six cycles for water.

## Introduction

The world population is anticipated to exceed nine billion by 2050^1,2^. This leads to increase in the demands for potable water and at the same time raises the production of wastewater. This continuing issue is often accompanied by ineffective wastewater management, deteriorating wastewater infrastructure facilities, and inadequate disposal schemes with limited or no treatment procedures^3^. Following these problems, a variety of chemicals that have the possibility of reaching surface waters are discharged daily into the environment^2,3^. Emerging contaminants (ECs) are an umbrella term for different substances that have garnered considerable concerns in the past two decades. These contaminants can be synthetic compounds or substances that occur naturally in minimal amount or no monitoring with a potential negative impact on human health and other organisms. These recalcitrant compounds consist of a variety of chemicals, such as pharmaceuticals, polycyclic aromatic hydrocarbons, personal care products, and pesticides^4,5^. The most common sources of ECs are industrial effluents, municipal sewage, wastewater treatment plants (WWTPs), household products, hospitals, landfills, and pharmaceutical production companies^6^. Among the aforementioned sources, wastewater treatment plant effluent is regarded as the major source of ECs. WWTPs are not designed to entirely remove ECs and their metabolites, so they can escape to the aquatic ecosystems in the discharged effluent. There are currently no guidelines or standards for the disposal and discharge of ECs in existing wastewater treatment plants^7^. In practice, conventional physical and chemical processes for the reducing of organic compounds from wastewater have several serious limitations, including insufficient purification, low efficacy, high costs, the formation of hazardous by-products, and application to a narrow concentration range^8,9^. Consequently, it is widely acknowledged that there is an urgent need to develop more effective, innovative, and environmentally friendly methods for wastewater remediation. Bioremediation or biodegradation, a prospective new area of investigation, has been utilized effectively for the elimination of ECs from water and wastewater^10^. Biodegradation methods have many benefits as opposed to the physiochemical methods, since they are more cost-effective, safer and less disruptive^11^.

Laccases (EC 1.10.3.2) are prevalent extracellular oxidoreductases in bacteria and plants. However, laccases derived from fungi, such as *Trametes versicolor, T. vilosa*, and *Cerrena unicolor*, are of considerable interest due to their high catalytic efficiency, cost-effectiveness, and accessibility^12^. The free-form enzymes are extremely challenging to be recovered from liquid samples^13^, and thus, can only be used one time for applications related to water treatment. This increases the cost of the process as more enzymes are required to be produced and purified^14^. Furthermore, the stability and activity of free enzymes are poor in complex matrices, such as heavily contaminated wastewater^15^. Enzymatic immobilization on a solid support is applied to enhance the stability and storage of enzymes^16^, which is an effective approach for addressing the aforementioned limitations. Several methods of laccase immobilization on nanomaterials, membranes, and fibers have been investigated via adsorption, encapsulation, and covalent bonding. Nevertheless, low-cost and environmentally friendly materials for the efficient removal of pollutants are still required^17^.

Activated carbon from agro-industrial waste, a carbon material derived from the pyrolysis of biomass material possesses advantageous characteristics such as a large specific surface area, excellent dispersibility, and biocompatibility for stable and high-load enzyme immobilization^18^. Several examples of agro-industrial residues serving as carriers have been reported, particularly for laccase with its potential in removing pollutants. According to Lonappan and coworkers^42^, diclofenac was biodegraded by immobilized laccase enzyme. The mature pig biochar had the highest laccase-binding capacity among the carrier supports used for enzyme immobilization, which included Pinewood and almond shell biochars. The citric acid pretreatment of biochars enhanced laccase binding. In addition, mature pig biochar immobilized laccase was able to completely remove diclofenac (500 μg/L) in two hours. In a separate study, Naghdi et al.^29^ investigated the bioremediation of carbamazepine (CPZ) by laccase enzyme immobilized on acid-treated pine wood nano-biochar. The immobilized biocatalytic system retained 70% of its initial activity after three cycles of reuse, removing 83% of CPZ from the contaminated water. Nanocellulose (NC) derived from quinoa husks (QS) was used as a carrier for laccase to eliminate two model dyes (malachite green (MG) and congo red (CR)) from water. After one hour, the system was more effective at decolorizing MG than CR (92% vs. 51%) when the dye concentration was 1000 mg/L. The system was able to decolorize concentrated dye solutions and demonstrated superior reusability (up to 83% dye removal after 18 cycles for MG) and exceptional efficacy from complex real textile effluents^82^. Imam and colleagues^63^ investigated the biodegradation of anthracene using laccase immobilized on the surface of acid-treated rice husk biochar. The results demonstrated that biochar-immobilized laccase treated with acid had a high immobilization yield of 66% and high operational stability. Additionally, this immobilized system completely degraded 50 mg/L anthracene in aqueous batch mode within 24 h. García‐Delgado and colleagues^83^ studied biochar (holm oak (Quercus ilex)) immobilized laccases, which were evaluated to remove 0.1 mmol/L of each antibiotic at a concentration of three tetracyclines and six sulfonamides. Utilizing *Pleurotus eryngii* laccase on biochar, high levels of activity yields (70.3%) and catalytic capacities (1405 IU/g) were attained. Only chlortetracycline was entirely eliminated in the presence of syringaldehyde, whereas immobilized-laccase/ABTS systems cleared all tetracyclines. Laccase immobilization on Spruce biochar (Sba) and Maple biochar (Mba) exhibited optimal immobilization at a pH value of 3, at a laccase concentration of 16 g/L for 8 h. The maple biochar with a high surface area and pore volume immobilized laccase more effectively than Sba, but the recovered activity was independent of the wood species employed. Immobilization enhanced the laccase's thermal stability and prevented it from enduring conformational changes^65^. Chen et al.^84^ degraded nine pesticides, but to assure maximum efficiency, they chose combined methods: biodegradation with laccase immobilized on two biosorbents. As supports, peanut shells and wheat straw were utilized, and syringaldehyde was added to the reaction system to enhance laccase's catalytic properties. Within three days, the pesticides in the water were subjected to removal tests. In a system where laccase was immobilized on peanut shells, 54.5% of the contamination could be degraded, whereas the reaction efficiency for wheat straw was 69.1%. In the following phase of the investigation, the source of contamination was altered. Using both the peanut shell system and the wheat straw system, biodegradation of pesticides in soil for seven days eliminated 20 to 92% of pollutants. Due to variations in pesticide resistance to enzymatic treatment, there is a significant disparity in results**.** Laccase was immobilized in biochar derived from avocado seeds and employed for the sorption and biotransformation of acetaminophen (ACT). After being treated with citric acid and glutaraldehyde, the biochar surface improved the enzyme and support's ability to bond. After the acid treatment, the surface area of biochar increased by approximately 12 times, and carbonyl groups were observed. The immobilized laccase maintained 50.7% of its activity in the biotransformation of ACT for up to seven reuse cycles. Additionally, the immobilized enzyme demonstrated storage stability for 30 d at 4 °C and 25 °C, retaining over 90% of its activity in the biotransformation of ACT^85^. Dos Santos et al.^86^ utilized corn cob as a green support for laccase immobilization and its application in the degradation of Remazol Brilliant Blue R (RBBR) dye with the goal of valorizing agro-industrial residues. The highest yields of immobilized protein (75%) and residual activity (40%) were obtained at pH 7.0 and an enzyme concentration of 0.1 g/mL, with an enzyme activity of 1854 U/kg. At 60 °C, more than 90% of the initial activity of the immobilized biocatalyst was still present. In 48 h, the immobilized enzyme degraded RBBR dye with a higher efficiency (64%), and the process improved in 72 h (75%).

Among the agro-industrial wastes that have not been used as a carrier for laccase immobilization to remove pharmaceutical contaminants is pomegranate peels (PPs). In 2017, global pomegranate production was predicted at 3.8 million metric tons^19^. Nonetheless, the actual quantity exceeds the estimated amount. The peel accounts for 50% of the pomegranate fruit^20^, thus the world produced approximately 1.9 million metric tons of peel in 2017. This large amount of biowaste can be turned into useful materials (e.g. adsorbents) for different applications such as water treatment. Consequently, with the treatment landscape pushing for environmentally friendly and carbon emission reduction options, it becomes imperative to use fossil-free resource materials such as PPs for bioremediation methods.

Yet, to the best of the authors’ knowledge, no studies have evaluated PPs as a carrier for laccase immobilization for the purpose of pharmaceutical removal and they only examined the PPs as an adsorbent^21–25^. Thus, the aim of this research was to evaluate the capacity of chemically functionalized PPs for dual applications as a laccase carrier and an adsorbent for the simultaneous removal and degradation of a range of emerging chemicals including diclofenac, amoxicillin, carbamazepine, and ciprofloxacin in water and real wastewater samples. The selection of these emerging pollutants was based on their frequent occurrence in water bodies and their potential environmental and health issues^26^. Two of these emerging pollutants, namely amoxicillin, and ciprofloxacin, are on the European Water Framework Directive's updated watch list^27^. Present study also examined the influence of operational factors such as pH, temperature, and laccase concentration on the adsorbability of laccase onto PPs. The study also assesses the stability and activity of adsorbed laccase on PPs over a number of operation cycles.

## Materials and methods

### Chemicals and biosorbent

The diclofenac (C_14_H_11_C_l2_NO_2_, CAS No.: 15307-86-5), amoxicillin (C_16_H_19_N_3_O_5_S, CAS No.: 26787-78-0), carbamazepine (C_15_H_12_N_2_O, CAS No.: 298-46-4), and ciprofloxacin (C_17_H_18_FN_3_O_3_, CAS No.: 85721-33-1) were purchased from Merck KGaA. The 2,2′-azino-bis (3-ethylbenzothiazoline-6-sulfonic acid) diammonium salt (ABTS) (98%), mineral acids, and *T. versicolor* laccase were obtained from Sigma-Aldrich. Pomegranate (*Punica granatum* L., family Punicaceae) peels (PPs) were obtained from a local shop free of charge in Iraq. Other chemicals used in this work were acquired from Sigma-Aldrich. The analytical-grade chemicals and reagents were used without additional purification.

### Activated carbon

As a biosorbent, pomegranate peels (PPs) were used for the elimination of emerging pollutants. The pomegranate peels as a raw carbonaceous material were cleaned with distilled water to eliminate dust and impurities, sun-dried for a week, and then heated in the oven at 105 °C for a day. The peels were ground by the electrical grinder and sieved to obtain particle sizes between 0.5 and 1 mm. The PPs were then soaked in distilled water for a day, and dried again at 105 °C. As reported in the literature^28,29^, the following procedures were employed for the purpose of preparing the activated carbon with some modifications: in a furnace, PPs were dried for 4 h at 105 °C, and then they were moved to a stainless-steel reactor and heated to 600 °C at a heating rate of 10 °C/min and maintained at this temperature for 0.5 h under a flow of nitrogen (150 mL/min) as a purging gas. After turning off the furnace, the nitrogen flow was maintained until the temperature fell below 200 °C. Afterward, three (3) grams of carbon ized material was mixed with 400 mL of sulfuric acid (5 M H_2_SO_4_) and nitric acid (5 M HNO_3_) (1:1 V/V) in an 800 mL boiling flask; the cocktail was agitated on a magnetic stirrer and refluxed at 80 °C for 4 h. Thereupon, the product was thoroughly washed with deionized water until the attainment of pH of 6–7. The material was then dried overnight at 80 °C in an oven with an air supply, pulverized using an agate mortar pestle to produce 100–125 µm particles, and kept in a sealed container for the subsequent investigation. The final product was labeled as modified pomegranate peels (MPPs).

### MPPs characterization

The synthesis of MPPs was characterized using a range of physical and chemical analyses. Fourier-transform infrared spectroscopy (FTIR) with a wavenumber range of 400 to 4000 cm^−1^ with 2 cm^−1^ resolution) with attenuated total reflection (ATR) was applied for studying the change in adsorbents’ structure (Nicolet™ iS™ 5 FTIR Spectrometer, Thermo Fisher, USA). Boehm titration was employed for quantifying functional groups^30^. Approximately 1 g of the material was shaken at room temperature for 72 h with 50 mL of 0.1 M solutions of NaOH, 0.1 M NaHCO_3_, 0.05 M Na_2_CO_3_, and 0.1 M NaOC_2_H_5_. The suspensions were then decanted and filtered. The solutions were then back titrated with 0.1 M solution of HCl. The basicity of the adsorbent was determined by an analogous procedure. The sample was contacted with 0.1 M HCl and for the titration, 0.1 M NaOH was used. The concentration of acidic sites on the adsorbent was determined by taking into account that NaHCO_3_ neutralizes carboxylic groups, Na_2_CO_3_ neutralizes carboxylic and lactonic groups, and NaOC_2_H_5_ neutralizes carboxylic, lactonic, phenolic, and carbonyl groups^31^. Scan electron microscopy (SEM) equipped with Energy Dispersive X-ray spectroscopy (EDS) was utilized for examining morphological characteristics and obtaining elemental mapping of adsorbents (FEI/Thermo-Fisher Apreo S LoVac SEM and AMETEK, USA). The samples were coated with gold to reduce sample charging and enhance SEM images. The pore size and surface area (S_BET_) were determined using Brunauer–Emmett–Teller (BET) method. The point of zero-charge (pH_PZC_) of MPPs was determined according to the drift method^32^. To properly assess the pH_pzc_, a series of solutions with initial pH values (pH_i_) ranging from 2.0 to 12 was set up with NaCl as the background electrolyte. Upon adding the desired amount of MPPs to each solution, the conical flasks were sealed with parafilm, and the suspensions were shaken at 150 rpm for 2 days. The final pH value (pH_f_) of each solution was then recorded, and the change (ΔpH) versus pH_i_ was plotted. XRD also tested for the MPPs.

### Proximate and ultimate analysis

The proximate analysis of PPs was conducted in accordance with ASTM standards. The ash content was determined after heating in a furnace at 550 °C for 4 h. The carbon, nitrogen, hydrogen, and oxygen elemental compositions of PPs were measured applying ASTM D3176 standard procedure and using an elemental analyzer Model EA 1108 (Carl Erba Instruments).

### Enzyme assay

The oxidation of ABTS as a substrate was utilized for determining the enzyme's activity. ABTS is an appropriate substrate for measuring the activity of laccase because it can be oxidized by laccase to create cation-radicals (ABTS^+^) that can be measured by spectrophotometrically. At typical assay conditions, the amount of enzyme that can oxidize one µmol of ABTS per minute was used to define one unit of enzyme activity. As reported in the literature^33^, for the free enzyme, 125 μL of diluted enzyme solution (2.5 mg/mL) was combined with 375 μL of newly made 0.1 mM ABTS in buffer solution at pH 4 for 2 min at 35 °C. The activity of LMPPs was determined by mixing 0.06 g in 6 mL of Mcilvaine buffer (0.12 M) pH 4 containing 0.5 mM ABTS and shaking it at 150 rpm for 15 min at room temperature. After centrifuging at a speed of 8000 rpm, an absorbance measurement was taken from a sample of a constant volume at regular intervals of three min. Spectrophotometric analysis of the color development was then performed at 420 nm using UV–vis (Shimadzu UV-2450, Tokyo, Japan) and applying ε = 3.6 × 10^4^ M^−1^ cm^−1^. The activity of LMPPs (laccase modified pomegranate peels) is expressed as U/g. Equations (1) and (2) were used for calculating immobilized and free enzyme activities, respectively^34^.

1 $$\mathrm{Immobilized \,laccase \,activity \,U}/\mathrm{g}=\frac{\Delta \mathrm{ab}\times {\mathrm{D}}_{\rm{f}}\times {\mathrm{R}}_{\rm{v}}}{\upepsilon \times {t\times \mathrm{M}}_{\rm{support}}}$$

2 $$\mathrm{Free \,laccase \,activiy \,U}/\mathrm{mL}= \frac{\Delta \mathrm{ab}\times {\mathrm{D}}_{\rm{f}}\times {\mathrm{R}}_{\rm{v}}}{\upepsilon \times \mathrm{t}\times \mathrm{v}}$$

where $\Delta$ab is the absorbance, D_f_ is the dilution factor, R_V_ is the reaction volume (mL), $\upepsilon$ (3.6 × 10^4^ M^−1^ cm^−1^) is the molar extinction coefficient, t is the reaction time (min), v is laccase amount (mL) and M_support_ is mass of the support on which laccase has immobilized (g).

### Immobilization

Two grams of MPPs were employed to immobilize the laccase enzyme in 20 mL of buffer solution (pH 4) containing 2.5 mg/mL of laccase. The cocktail was stirred for 6 h at 35 °C and 150 rpm in a 50 mL tarson tube (Polypropylene/Spinwin Conical-Bottom). The cocktail was then centrifuged for 5 min at 8000 rpm. The immobilization yield was determined by dividing the difference between the enzyme activity in the supernatant prior to and after immobilization by the enzyme activity in the supernatant prior to immobilization^35^. The MPPs were washed twice with a 5 mL buffer solution (pH 4) to remove the excess enzyme, and the residual enzyme activity was also evaluated. The final product marked as LMPPs was refrigerated for further examination.

### pH, temperature, laccase dose, and storage stability

Regarding the pH stabilization, 100 μL of free laccase (2.5 mg/mL) and 20 mg of LMPPs were added to separate tubes containing 4 mL of buffer solutions (pH range: 3 to 8) and mixed at 150 rpm at 35 °C for 6 h. The residual laccase activity of both unbound and bound samples was tested. For thermal stability, a protocol identical to that for pH stabilization was followed, with the exception that samples were held at varying temperatures (10–60 °C) for 6 h at a fixed pH of 4. The optimum laccase dosage was determined by adding various laccase concentrations (ranging from 0.5 to 3 mg/mL) to 20 mg of LMPPs in separate tubes containing 4 mL of buffer solution (pH 4) and incubating them for 6 h at 35 °C and 150 rpm. For storage stability, free and immobilized enzyme samples were kept for up to two months at 4 and 25 °C, respectively, and the residual activity was evaluated every week.

### Reusability tests

LMPPs (100 mg) was mixed with 2 mL of buffer solution (pH 4) comprising 0.5 mM ABTS and kept for 10 min at 35 °C and 150 rpm. The sample was centrifuged for 3 min at 8000 rpm, and the concentration of transformed ABTS in the supernatant was determined. Six cycles of washing with Milli-Q water and decanting were performed on LMPPs.

### HPLC analysis

The concentration of pollutants was determined using high-performance liquid chromatography (HPLC) type Merck-Hitachi D-7000^36^. With gradient elution, the HPLC column used was a Zorbax SB-Aq of 150 mm length and 4.6 mm in diameter, with 5 µm particle size (Agilent, Santa Clara, CA, USA). The volume of the injected sample was 10 µL. The mobile phase was composed of 0.1% trifluoroacetic acid in Milli-Q water as eluent A and methanol as eluent B. The gradient program comprises 0–1 min of 40% mobile phase B and 1–5 min of a gradient to 100% mobile phase B. This lasted for up to eight minutes. Following this, the gradient reverted to its original state. The flow rate was set at 1 mL/min. Under these conditions, the compounds' calibrations were linear between 5 and 100 g/mL.

### Removal test and long-term performance

In batch experiments using Milli-Q water and secondary effluent from a wastewater treatment plant, the effectiveness of LMPPs for removing emerging contaminants from an aqueous solution was examined. Before processing the wastewater samples, they were filtered using Whatman cellulose filter paper (47 mm circle) and the pollutants were spiked in the samples. In the tests, 100 mg of LMPPs was added to 40 mL of a pollutant mixture (50 mg/L, from each) and mixed at 150 rpm at room temperature. Samples of 2 mL were collected every half an hour, and then a 24-h sample was collected. The purpose of this step was to determine when the system reach equilibrium. Consequently, the system attained equilibrium at 120 min after evaluating the whole measurements. Based on the initial and final aqueous phase concentrations, the removal efficiency was calculated. The LMPPs were cleaned with Milli-Q water and dried, and the cycle continues (every 120 min). The removal of pollutants by MPPs (without laccase) due to adsorption was also examined to understand the contribution of physical removal and degradation. This was accomplished by adding 100 mg of MPPs to 40 mL of a pollutant mixture (50 mg/L) and mixing at 150 rpm at room temperature. Samples of 2 mL were withdrawn every 30 min for two hours.

### Enzyme kinetic, adsorption isotherms, kinetics, and thermodynamics

The Michaelis–Menten kinetic parameters of free and immobilized laccase were measured with ABTS at different concentrations in buffer solutions under optimal conditions, and the estimated kinetic parameters (K_m_ and v_m_) were determined using the equations below.

3 $$\mathrm{v}=\frac{{\mathrm{v}}_{m }[\mathrm{S}]}{{\mathrm{K}}_{m}+[\mathrm{S}]}$$

Where v is the reaction velocity (mM/ min), v_m_ is the maximum reaction velocity (mM/ min), [S] is the substrate concentration (mM), and K_m_ is the Michaelis–Menten constant (mM). Table 1 shows the models used for studying the adsorption isotherms, kinetics, and thermodynamics.

**Table 1:** Adsorption isotherms, kinetics, and thermodynamics.

Isotherms, kinetics, and thermodynamics	Equation	References	Parameters
Langmuir	$\frac{1}{{q}_{e}}=\frac{1}{{q}_{m}}+\frac{1}{{q}_{m}{\times K}_{L}}\times {{(C}_{e})}^{-1}$	^37^	q_m_ = maximum adsorption on MPPs (mg/g), K_L_ = Langmuir constant (L/mg)
Freundlich	${\mathrm{Log} q}_{e}=\frac{1}{n}\mathrm{Log }{C}_{e}+{\mathrm{Log }K}_{f}$	^38^	q_e_ = chemicals absorbed per unit adsorbent at equilibrium (mg/g) C_e_ = equilibrium adsorbate concentration (mg/L) K_f_ and n = Freundlich adsorption isotherm constants
Pseudo-first-order	$\mathrm{ln}\left({q}_{e}-{q}_{t}\right)=\mathrm{ln}{q}_{e}-{K}_{1}t$	^39^	q_e_ = chemicals adsorbed at equilibrium (mg/g) q_t_ = chemicals absorbed at time t (mg/g) K_1_ = pseudo-first-order constant rate (min^−1^)
Pseudo-second-order	$\frac{t}{{q}_{t}}=\frac{1}{{K}_{2}{q}_{e}^{2}}+\frac{t}{{q}_{e}}$	^40^	K_2_ = pseudo-second-order constant rate (g mg^−1^ min^−1^)
Thermodynamics	$\Delta G ^{\circ}=-\mathrm{R}\times \mathrm{T}\times \mathrm{ln}{(K}_{L})$ ${K}_{L}=\frac{{q}_{e}}{{C}_{e}}$ $\mathrm{ln}{(K}_{L})=\frac{-\Delta H ^{\circ}}{\mathrm{RT}}+ \frac{\Delta S ^{\circ}}{\mathrm{R}}$ $\Delta G ^{\circ}={\Delta H}^\circ -\mathrm{T}\times {\Delta S}^\circ$	^41^	ΔG° = Gibbs free energy, ΔS° = standard entropy ΔH°= standard enthalpy T = temperature (K) R = the universal gas constant (8.314 J/mol K)

### Quality control

All experiments were conducted in triplicate, and results are presented as mean and standard deviation. The data were evaluated using the Kolmogorov–Smirnov test and results with p < 0.05 were considered statistically significant. Blank samples were also used in each experiment.

### Complies with international, national and/or institutional guidelines

Experimental research and field studies on plants (either cultivated or wild), including the collection of plant material, comply with relevant institutional, national, and international guidelines and legislation. Experimental studies were carried out in accordance with relevant institutional, national or international guidelines or regulations.

## Results and discussion

### MPPs characterization

**Figure 1:**
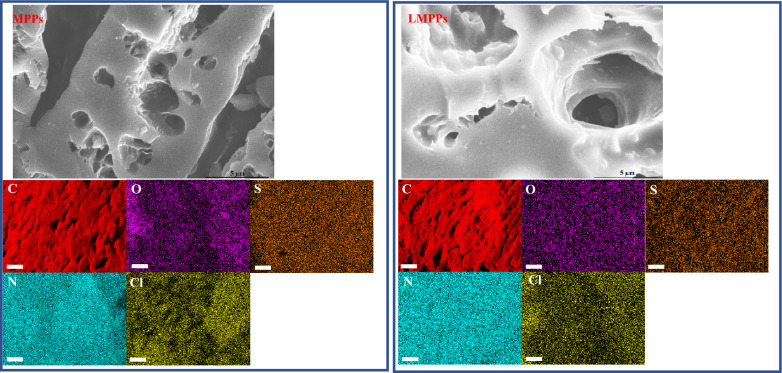
SEM and EDS for MPPs and LMPPs. EDS scale is 1 µm.

Figure 1 depicts SEM images and EDS elemental maps for MPPs and LMPPs. There were no discernible surface texture alterations following the immobilization of laccase. Due to their diminutive size (60–90 KDa), which corresponds to a particle size of less than 5 nm, laccases may not be identifiable by SEM at these magnifications. Micrographs with a 1 μm magnification have difficulty capturing this dimension^42^. Meanwhile, after the immobilization, the surface of LMPPs appeared smoother, most likely as a result of the laccase coating on the activated carbon surface^43^. Enzyme immobilization in magnetic biochar nanoparticles has been correlated with the smoothness of the surface^44^. Similar findings were observed in SEM images described in other investigations^34,36,45^. Activated MPPs with inorganic acids exhibited a more heterogeneous surface with deeper pores, which could be ascribed to the acid's ability to remove impurities from the pores^34^. In a similar study, Lonappan and colleagues^46^ found that biochar subjected to citric acid exhibited more porous structures. The MPPs and LMPPs surfaces contain C, O, S, N, and Cl, as shown in Fig. 1. The presence of oxygen-containing groups on the surface was indicated by the presence of oxygen in MPPs and LMPPs EDS mapping. Element (N) increased in LMPPs relative to MPPs (Fig. S1), indicating the presence of laccase on the surface of the adsorbent^34,36^. However, the decrease in S concentration suggests that chemical reactions might have occurred during the treatment. Figure S2 depicts the XRD patterns of MPPs. A broad band between 20° and 30° corresponds to amorphous carbon. This also indicates that synthesized MPPs has more amorphous pore walls, a larger superficial area, and low crystallinity, presumably as a consequence of the high-temperature carbon pyrolysis.

**Figure 2:**
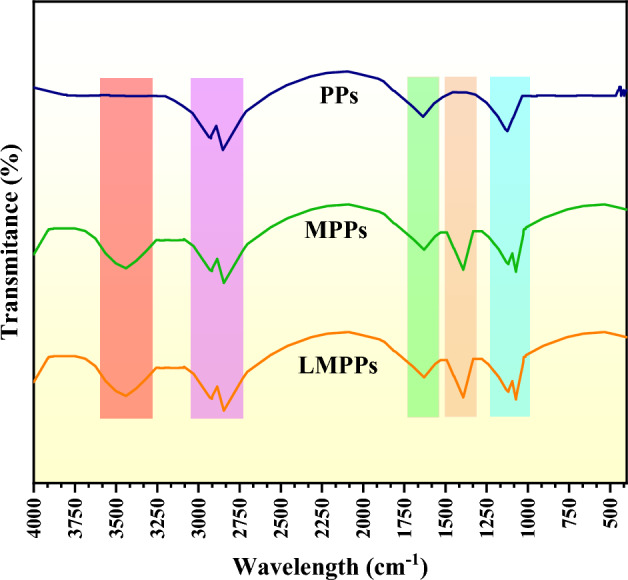
FTIR spectra for PPs, MPPs and LMPPs.

The FTIR spectra of the adsorbents are shown in Fig. 2. PPs, MPPs, and LMPPs exhibited CH_2_ stretching bands at 2922 and 2846 cm^−1^ that were asymmetrical and symmetrical, respectively. PPs, MPPs, and LMPPs contained a carbonyl phenolic groups indicated by the peak at 1632 cm^−1^ and at 1118 cm^−1^ bands, respectively. MPPs and LMPPs exhibited C-O stretching at 1382 cm^−1^ as a result of chemical treatment by inorganic acids^47^. The presence of enzyme had a very subtle impact on the FTIR spectra of modified adsorbents as demonstrated by a slight increase and broadening of the peak at 3436 cm^−1^. This peak corresponds to laccase O–H and N–H bonds overlapping. Peak at 1632 cm^−1^ is expected to be associated with N–H stretching vibration and the amide bond existing in the laccase protein, which is regarded as an indicator of laccase protein content^48^.

The formation of functional groups on the surface of carbonaceous substances offers optimal anchoring sites for the physical attachment and covalent bonding of enzymes to their surface^49^. Because carboxylic groups are easily produced through oxidizing treatment, they can undergo a variety of chemical reactions^50^. Additionally, it has been reported that the presence of carboxyl groups on the surface of a carrier can enhance the immobilization of enzymes^51^. Thus, according to the results of the Boehm titration (Table 2), MPPs has a total acidity of 7.02 meq/g and are composed of carboxylic (1.27 meq/g), carbonyl (2.31 meq/g), and phenolic (3.44 meq/g) groups. The surface functional groups increased about 2.6-fold for the acidic groups and decreased by 0.08 meq/g for the total surface basicity. This increase was expected as during the activation process more acidic materials formed from the interaction between inorganic acids and the carbon precursor. The quantification of functional groups with Boehm titration agrees well with the qualitative FTIR results. These findings are in line with Zhang et al.^52^ who found a similar trend after activation with an increased acidic group and a decrease in the basic one. Another study used sonication in a mixture of H_2_SO_4_ and HNO_3_ acids, to modify single-walled carbon nanotubes. It was discovered that 14 h of sonication contributed to cut nanotubes and increasing the concentration of COOH groups from 0.91 to 6.4 mmol/g^53^.

**Table 2:** Boehm titration (meq/g).

Functional groups	PPs	MPPs
Carboxylic groups	0.42	1.27
Carbonyl groups	0.93	2.31
Phenolic groups	1.28	3.44
Total surface acidity	2.63	7.02
Total surface basicity	0.73	0.65

Specific surface area (S_BET_) and total pore volume (V_total_) of PPs, MPPs, LMPPs were 47.6 m^2^/g and 0.163 cm^3^/g, 1249.1 m^2^/g and 0.831 cm^3^/g, 232.7 m^2^/g and 0.271 cm^3^/g, respectively, as shown in Table 3. Comparing the S_BET_ from Table 3, it can be revealed that the S_BET_ for PPs increased around 26.24 times after the modification. However, S_BET_ for MPPs decreased by 81.40% after the immobilization step to become 232.7 m^2^/g for LMPPs. This larger reduction in the surface area provided quantitative proof for the immobilization of laccase via surface adsorption. These obtained surface area and pore volume in this study are in the same range as those reported in the literature. For instance, Hadi and colleagues^54^ prepared magnetized activated carbon synthesized from pomegranate husk and reported V_tot_ and S_BET_ of 0.75 cm^3^/g and 1363.4 m^2^/g, respectively. Similarly, Taheri and co-workers^55^ obtained V_tot_ and S_BET_ of 0.83 cm^3^/g and 1576 m^2^/g, respectively, for their activated biochar made of the same raw materials but different activation methods using zinc chloride followed by hydrochloric acid treatment. Another study used microwave-induced and KOH-activation procedures for preparing pomegranate peel activated carbon and their measured S_BET_ and V_tot_ were 941.02 m^2^/g and 0.470 cm^3^/g, respectively^56^. The S_BET_ reduction following enzyme immobilization was also reported in numerous previous works. For instance, He and co-workers (2006)^57^ conducted an investigation to immobilize lipase on mesoporous silica which revealed a surface area reduction of up to 89.1%. Badgujar and colleagues^58^ tested immobilized lipase on a polymeric composite and found that the N-accessible surface decreased by 45%, from 0.804 to 0.437 m^2^/g. Similarly, Pirozzi and collaborators^59^ entrapped lipase in a ZrO_2_ porous structure and reported that the support's surface area decreased by 31%, from 316 to 229 m^2^/g.

**Table 3:** S_BET_, total pore volume, micropore volume, and mesopore volume of PPs, MPPs, LMPPs.

	S_BET_ ( m^2^/g)	V_tot_	cm^3^/g	V_mes_
PPs	47.6	0.163	0.057	0.106
MPPs	1249.1	0.831	0.313	0.518
LMPPs	232.7	0.271	0.097	0.174

**Figure 3:**
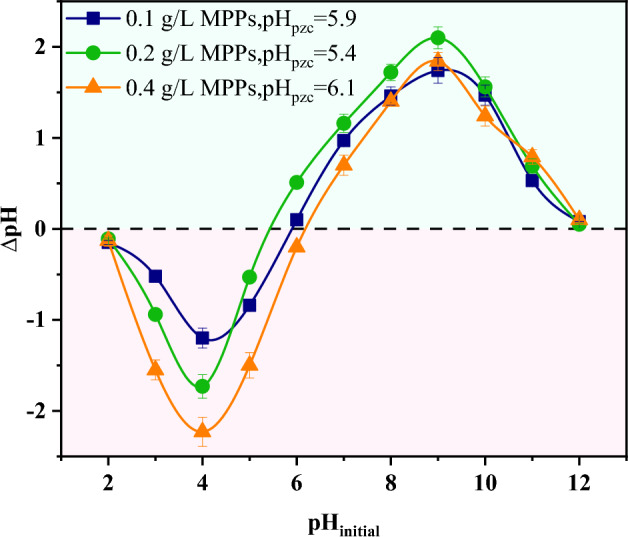
pH_pzc_ of different MPPs dosages.

Figure 3 depicts the impact of different MPPs dosage on the pH_pzc_. The pH_pzc_ was determined using the drift method, owing to its simplicity and inexpensive cost. The pH_pzc_ was obtained from the intersection of the pH (pH_final_ − pH_initial_) and pH_initial_. According to Fig. 3, the pH_pzc_ values of MPPs at doses of 0.1, 0.2, and 0.4 g/L adsorbent were 5.9, 5.4, and 6.1, respectively. The pH_pzc_ indicates that the MPPs has a positive surface charge at pH values less than 5.8 (the average value for three doses), demonstrating that acidic groups predominate on the surface of the MPPs and a negative surface charge at pH values greater than 5.8. In general, increased dosing of adsorbent caused the pH to drift toward point of zero charge (PZC)^32^, and thus the value of the PZC is dependent on the solid/liquid ratio. The different pH_pzc_ values have been also reported in the scientific literature. For instance, Umpierres and co-workers^60^ investigated microwave applications for carbon-based adsorbents derived from *Astrocaryum aculeatum* seed and observed that the pH_pzc_ value ranged between 4.44 and 6.71. Lima and colleagues^61^ observed that the pH_pzc_ value for carbon-based compounds synthesized from Brazilian nutshells ranged from 5.86 to 6.31. This variation in pH_pzc_ values is probably related to the raw biomass, activation and preparation methods for activated carbon.

The physical and chemical properties of PPs are shown in Table 4. PPs consist of carbon (46.23%), oxygen (42.83%), hydrogen (5.43%), nitrogen (3.47%), and sulfur (2.03%). These compositions are comparable to other PPs structure reported in the literature. For instance, Ben-Ali and co-workers^25^ found that the PPs composition is carbon (43.13%), oxygen (48.15%), hydrogen (7.17%), nitrogen (0.66%), and sulfur (0.89%). Siddiqui et al.^62^ also reported that the PPs main three elements were carbon (44.5%), oxygen (37.8%), and hydrogen (5.28%).

**Table 4:** PPs physical properties and elemental composition.

Parameters (%)	(Wt%) ± SD
Moisture content	7.89 ± 1.07
Volatile matter	62.43 ± 3.35
Ash	9.92 ± 0.95
Fixed carbon	32.46 ± 3.10
C	46.23 ± 2.11
O	42.83 ± 3.24
H	5.43 ± 1.16
N	3.47 ± 0.04
S	2.03 ± 0.17
H/C	0.12
O/C	0.93
N/C	0.08
S/C	0.04

### Optimization of laccase immobilization parameters

**Figure 4:**
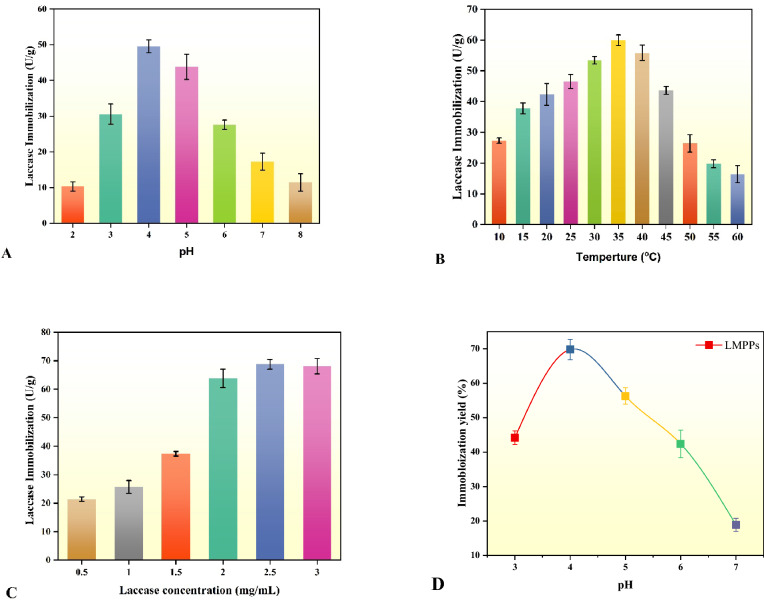
The influence of varying pH (**A**), temperatures (**B**), and laccase concentrations (**C**) on the immobilization and yield (**D**).

To achieve optimal enzyme immobilization, the conditions were optimized by varying pH, temperature, and enzyme concentration. The pH of the solution influences the stability of the laccase, thus, rendering it an essential variable in the immobilization procedure. Figure 4A presents the tested pH range (2 to 8) on the LMPPs. At pH 2, laccase immobilization was lower owing to a loss of enzyme activity (10.34 U/g), whereas, at pH 4, optimal enzyme immobilization was obtained (49.54 U/g). With increasing pH more than 4, laccase immobilization on MPPs dropped sharply. At a pH less than the isoelectric point of 5.8 (the average value), the net charge on MPPs is positive, which improves the electrostatic attraction between the carrier and the negatively charged laccase^63^. Temperature is also an essential variable in laccase immobilization, as enzymes are susceptible to heat by nature and are only functioning within specific temperature intervals. Figure 4B illustrates the temperature on the immobilization in the range of 10–60 °C. Laccase immobilization increased from 27.29 to 59.89 U/g as the temperature raised from 10 to 35 °C, which could be attributed to the higher rate of enzyme adsorption on MPPs. Following the optimum immobilization temperature (35 °C), the laccase immobilization decreased to 16.4 U/g at 60 °C as a result of a possible decrease in enzyme viability as the temperature increased^64^. Laccase concentration is vital for enzyme immobilization on MPPs. As shown in Fig. 4C, raising the enzyme concentration from 0.5 to 0.5 mg/mL enhanced enzyme immobilization from 21.40 to 68.76 U/g. Nevertheless, an additional increase in laccase concentration had no discernible effect on the immobilization of the laccase. This could be ascribed to the enzyme's ability to occupy available sites on the surface of MPPs. Enzyme immobilization on any material is contingent upon both the surface characteristics of the carrier and the immobilization settings. On the basis of a single-variable technique, the optimal immobilization settings were determined to be pH 4, a temperature of 35 °C, and an enzyme concentration of 2.5 mg/mL, which led to an immobilization yield of 69.8% (Fig. 4D). Increased immobilization yield was attributable to the optimal setting and modification of PPs with abundant carbonyl groups^42^. A lignocellulosic biochar immobilization yields of 64.23% has also been reported^65^.

### Laccase stability

**Figure 5:**
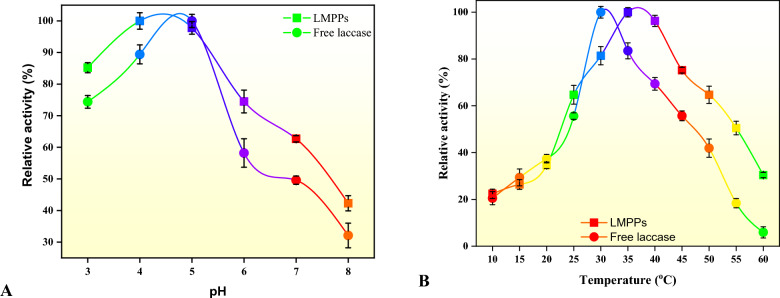
Stability of free and immobilized enzymes with regard to (**A**) pH and (**B**) temperature.

The pH of the solution may alter the ionization state of the amino acids in the enzyme, consequently affecting its structure, activity, and possibly leading to its denaturation^66^. Thus, the activity characteristics of free and immobilized laccase on MPPs were examined using activity assays at a range of pH values, as depicted in Fig. 5. Both free and immobilized laccase exhibited the highest levels of activity at pH 5 and 4, most likely due to the limited mobility of laccase caused by ionic interactions between the enzyme and MPPs^67,68^. In addition, LMPPs had a wider range of operating pH values than the free laccase, indicating that the LMPPs was more efficient than the free laccase under a variety of pH settings. The effect of temperature on the activity profiles of LMPPs and free laccase was also explored. According to Fig. 5, free laccase exhibited the highest enzyme activity at 30 °C, whereas immobilized laccase exhibited the maximum activity at 35 °C. Even at higher temperatures of 50 °C, 55 °C, and 60 °C, the LMPPs were more efficient than free laccase, with relative enzyme activities of 64.7%, 50.5%, and 30.4%, respectively. The high activities of LMPPs shown in these high temperatures could be because of the multipoint attachment between the laccase molecules and the MPPs, as well as the improved substrate diffusion capability at higher temperatures^42^. This also demonstrated that MPPs can assist in maintaining the activity of laccase, most likely because they could protect the conformational structure of laccase under various environmental circumstances^69^.

### Storage stability and operational cycles

**Figure 6:**
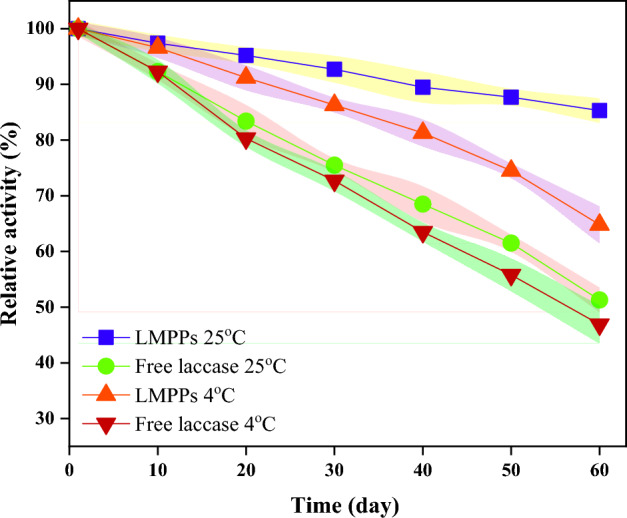
Stability of free and immobilized laccase (LMPPs) with two different temperatures 4 °C and 25 °C.

The storage stability of immobilized laccase is a crucial determinant of its applications in industry such as wastewater treatment. The storage stability of free and immobilized laccase with two different temperatures (4 °C and 25 °C) was compared over two months as illustrated in Fig. 6 with error bars presented as bands. After one month, immobilized laccase retained approximately 92.7% and 86.3% of its initial activity at 25 °C and 4 °C, respectively. In addition, after two months of storage, over 85.3% and 64.8% of the LMPPs initial activity were retained, at 25 °C and 4 °C, respectively. Comparatively, laccase retained approximately 75.5% and 72.7% of its catalytic properties after one months of storage at 25 °C and 4 °C, respectively. Upon completing 2 months of incubation at 25 °C and 4 °C, the free laccase activity decreased dramatically to 51.3% and 46.9% of its initial activities, respectively. The immobilized laccase is significantly more stable during storage, in contrast to the free one. This could be due to the stabilizing impact of the laccase's three-dimensional structure after immobilization and the protective role of the carrier, which restricts conformational changes of the biocatalyst^70^.

**Figure 7:**
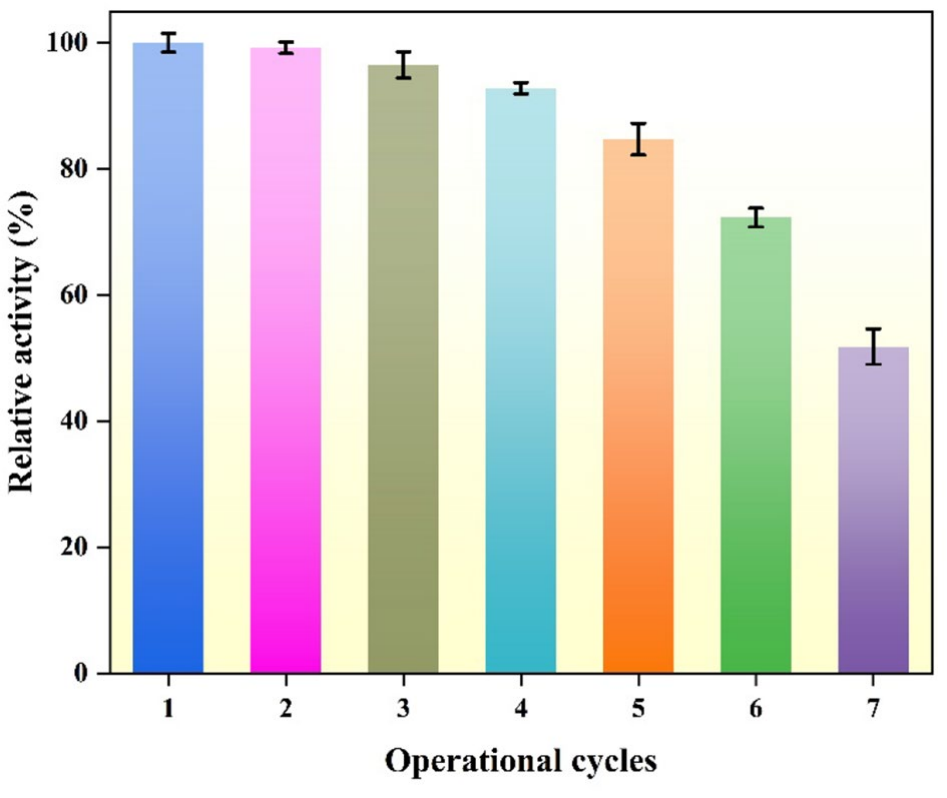
Stability results of immobilized laccase over seven operational cycles.

Among the benefits of immobilized enzyme is its reusability, which can also result in decreased costs and permit its application in continuous bioreactor operations^71^. To determine reusability, seven cycles of reaction between immobilized laccase and ABTS were examined for operational stability (Fig. 7). After seven cycles, immobilized laccase had lost approximately 48.2% of its initial activity. Since more than half of the initial laccase activity was still present after seven cycles of catalysis, laccase bound on MPPs appears to be reasonably stable. However, the following factors presumably led to the decline in activity: (1) a portion of immobilized laccase with weak binding was desorbed from the LMPPs during the rinsing procedure. Thus, only the laccase that is more firmly attached to the carrier is retained for subsequent cycles. (2) enzyme activity may be diminished throughout storage^71^. In this investigation, the operational stability experiment took a week to be completed, because the reaction between immobilized laccase and ABTS was a slow process and consistently decreased as the measurements were performed. This was also reported in another study that measured the operational cycles for immobilized laccase on different types of wood biochar^65^. Cristóvão and colleagues^35^ immobilized laccase on green coconut fibers and found that after five cycles of ABTS oxidation, their biocatalyst lost 30% of its initial activity. Overall, it is obvious from reusability experiments that laccase immobilization on MPPs substantially improves the operational stability of the enzyme.

### Laccase kinetics

**Figure 8:**
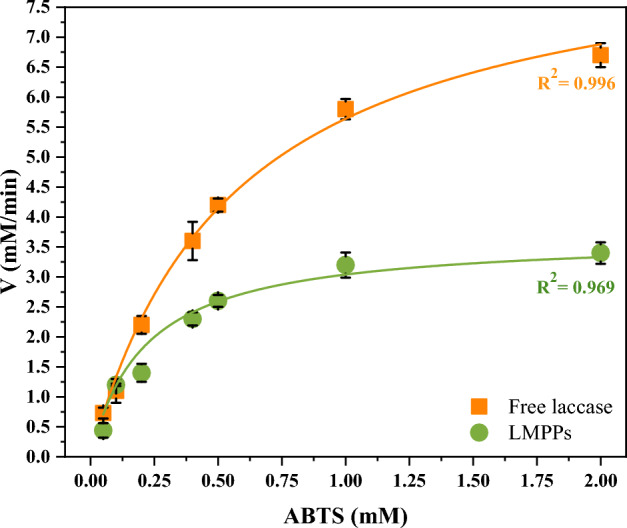
Michaelis–Menten fitting model for LMPPs and free enzyme.

The Michaelis constant (K_m_) is an essential factor for determining the affinity of laccase with its substrate, whereas the V_max_ value indicates the maximal reaction velocity. A lower K_m_ value indicates that the substrate has a higher binding affinity. The effect of substrate concentration (ABTS) between 0.05 and 2.0 mM on laccase immobilized activity at pH 4 was examined (Fig. 8). According to the Michaelis–Menten hyperbolic trend, a rise in substrate concentration results in a rise in the enzymatic reaction rate. The findings demonstrated that the immobilized laccase had a lower K_m_ value (0.21 mM) than the free laccase (0.56 mM), demonstrating the highest affinity towards ABTS and that the immobilization procedure could potentially have a positive effect on the substrate-enzyme interaction. This could be due to mass transfer limitations between the substrate and the surface of LMPPs. The lower V_max_ value of immobilized laccase (3.68 mM/min) compared to that of free laccase (8.82 mM/min) could be a result of the immobilized laccase's reduced flexibility and fewer accessible active sites. Other investigations have achieved a similar outcome^72^ demonstrating a decreased substrate affinity due to diffusional limitations and decreased enzyme flexibility after immobilization.

### Adsorption isotherms and kinetics

**Figure 9:**
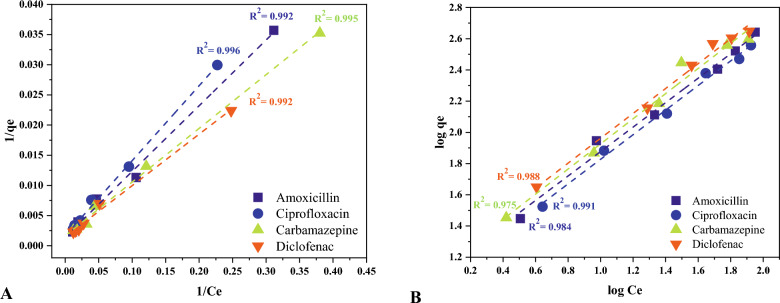
Langmuir (**A**) and Freundlich (**B**) isotherm models fittings.

**Figure 10:**
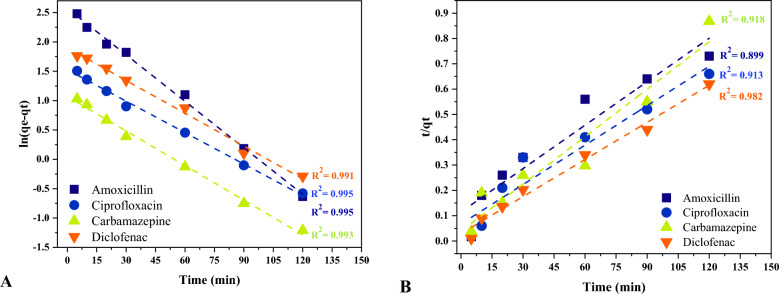
Adsorption kinetics of pharmaceuticals (**A**) pseudo-first-order and (**B**) pseudo-second-order.

**Figure 11:**
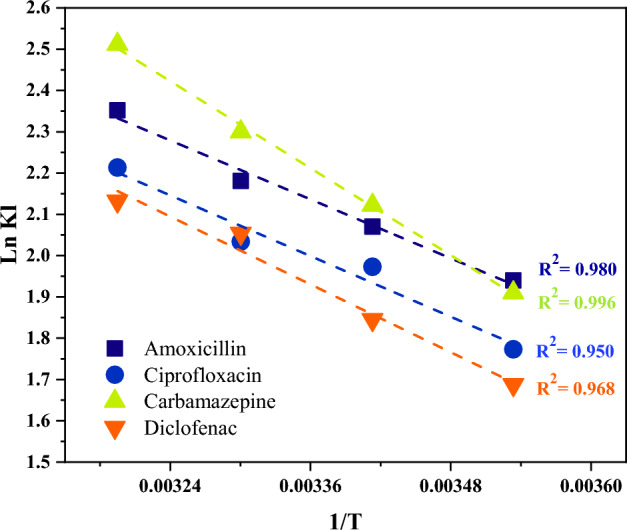
lnK_l_ vs 1/T for MPPs.

The results of applying experimental data to the Langmuir and Freundlich isotherm models are depicted in Fig. 9. Langmuir isotherm model suits the compounds' adsorption data better than the Freundlich model with correlation coefficients varying from 0.992 (amoxicillin and diclofenac) to 0.996 (ciprofloxacin). This suggests that the pharmaceutical molecules might have formed a monolayer on the surface of MPPs. The sorption capacity (q_m_) was calculated between 598.80 and 704.22 mg/g for ciprofloxacin and amoxicillin, respectively. As shown in Table 5 which illustrates Langmuir, Freundlich, Pseudo-1st-Order Model, and Pseudo-2nd-Order Model parameters, the separation factor (RL) values of the compounds between 0 and 1 indicates favorable adsorption. The Freundlich constant (n) is another factor applied in the adsorption literature to examine the favorability of the process. When the value of n is less than one, the adsorption is deemed preferable^73^. The heterogeneity factor (1/n) is used as an indicator for measuring the difference in the energy distribution of the adsorption sites. In this study, the 1/n values for the compounds were close to 0.8 indicating a heterogenous adsorption of the treated pharmaceuticals. Figure 10 depicts the pseudo-first and second-order models for the adsorption of chemicals. The experimental correlation coefficients best suit the pseudo-first-order kinetic model, which ranged from 0.984 (carbamazepine) to 0.995 (amoxicillin and ciprofloxacin). This demonstrates that physisorption governs the compounds adsorption rate onto MPPs. Figure 11 depicts the thermodynamic parameters plot of the compounds on MPPs at various temperatures (283, 293, 303, and 313 K). Table 6 shows the thermodynamic parameters of the pharmaceuticals’ adsorption onto MPPs. The G^o^ values reduced as temperature increased, indicating that temperature has a positive effect on the effectiveness of adsorption. Negative G^o^ values determined at four temperatures demonstrate that the compounds spontaneously adsorb onto MPPs. The calculated H° values were positive, suggesting that the adsorption was endothermic. Finally, positive S° values implies that the adsorption process is stable and random^74–78^.

**Table 5:** the parameter of Langmuir, Freundlich, Pseudo-1st-Order Model and Pseudo-2nd-Order Model.

Langmuir	qmax (mg/g)	K_L_	R_L_	R^2^
Amoxicillin	704.22	0.013	0.939	0.992
Ciprofloxacin	598.80	0.013	0.937	0.996
Carbamazepine	606.06	0.019	0.915	0.995
Diclofenac	704.21	0.017	0.923	0.992
Freundlich	1/n	K_f_	R^2^	
Amoxicillin	0.78	12.38	0.984	
Ciprofloxacin	0.78	10.93	0.991	
Carbamazepine	0.80	13.20	0.975	
Diclofenac	0.79	14.58	0.988	

Pseudo-1st-order model	qe (mg/g)	K1	R^2^	
Amoxicillin	13.08	1.8E−05	0.995	
Ciprofloxacin	4.59	1.2E−05	0.995	
Carbamazepine	2.91	1.3E−05	0.984	
Diclofenac	6.69	1.2E−05	0.991	

Pseudo-2nd-order model	qe (mg/g)	qe^2^	K2	R^2^
Amoxicillin	1.7E+02	3.0E+04	2.8E−04	0.899
Ciprofloxacin	2.0E+02	4.0E+04	3.7E−04	0.913
Carbamazepine	1.6E+02	2.5E+04	1.0E−03	0.918
Diclofenac	2.5E+02	6.2E+04	5.5E−04	0.982

**Table 6:** Thermodynamic parameters of emerging pollutants adsorption on MPPs.

Pharmaceutical compound	Temperature (K)	Kl	ΔG°	ΔH°	ΔS°	R^2^
Amoxicillin	283	6.95	−4.56	9.89	50.99	0.980
	293	7.92	−5.04			
	303	8.85	−5.49			
	313	10.50	−6.12			
Ciprofloxacin	283	5.88	−4.17	10.18	50.83	0.950
	293	7.19	−4.80			
	303	7.64	−5.12			
	313	9.14	−5.75			
Carbamazepine	283	6.75	−4.49	14.60	67.44	0.996
	293	8.34	−5.16			
	303	9.97	−5.79			
	313	12.32	−6.53			
Diclofenac	283	5.30	−3.96	11.38	54.30	0.968
	293	6.28	−4.49			
	303	7.78	−5.17			
	313	8.43	−5.54			

Table S1 shows the residual sum of square (or error sum of square, SSE) for the isotherm and kinetic models. A low SSE demonstrates that the model fits the data which in this work followed the Langmuir model and pseudo-first-order.

### Emerging pollutants removal from water and wastewater in short and long-term tests

**Figure 12:**
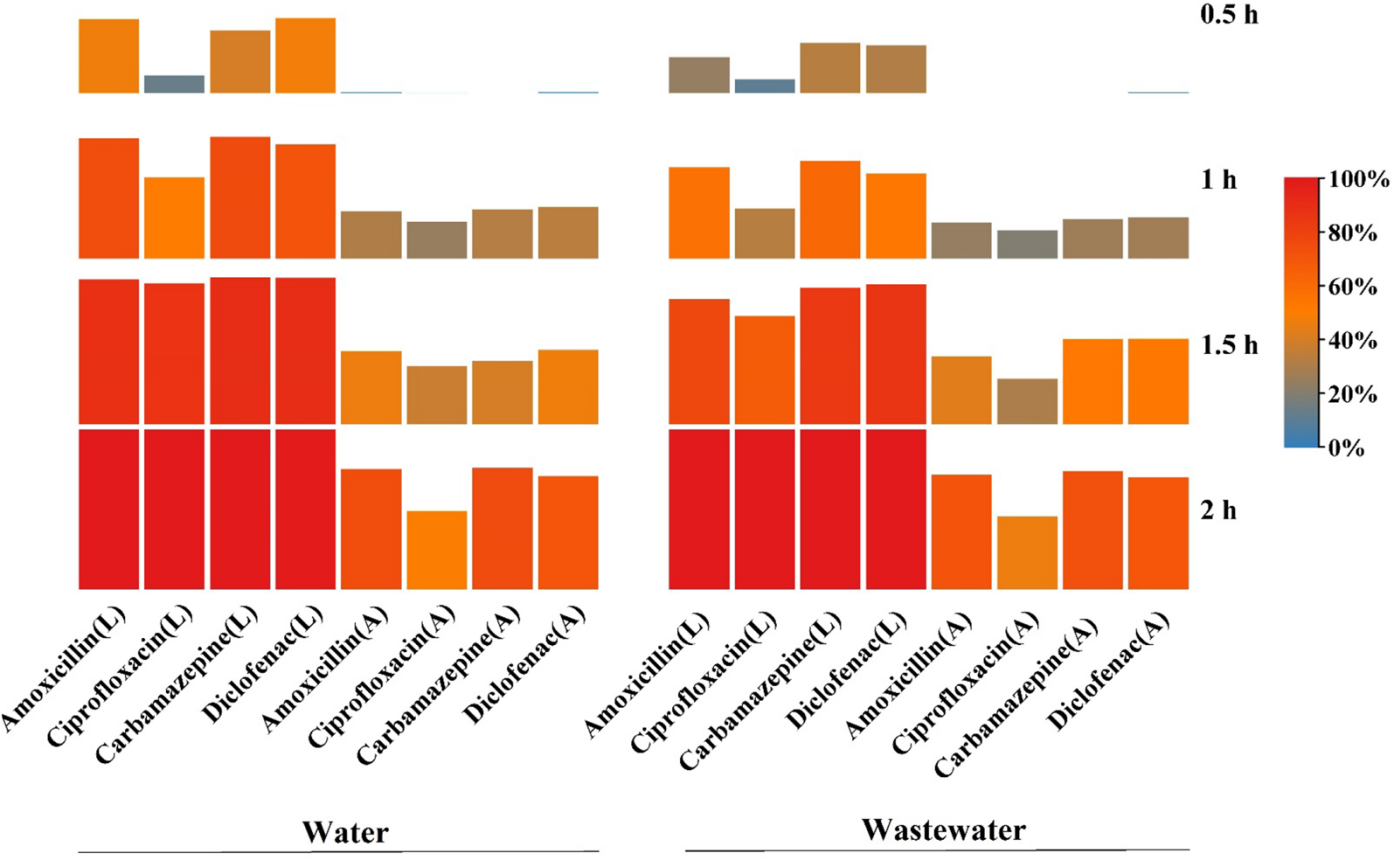
Emerging pollutants removal (%) with by LMPPs represented by a letter (L) and MPPs represented by (A), SD > 3.54%.

This study assessed the biodegradation of amoxicillin, ciprofloxacin, carbamazepine, and diclofenac in water and wastewater samples. Table S2 shows the secondary wastewater effluent characteristics. The adsorption of the target pollutants onto MPPs was examined through controlled experiments. The effectiveness of removing amoxicillin, ciprofloxacin, carbamazepine, and diclofenac from water and wastewater using LMPPs and MPPs over a two-hour period is depicted in Fig. 12. The letter A represents removal by adsorption only, whereas the letter L implies removal by adsorption and enzymatic degradation.

In both water and wastewater, LMPPs performed better than MPPs. Within 1.5 h, the removal efficiencies for LMPPs and MPPs ranged from 86.8% (ciprofloxacin) to 90.4% (carbamazepine) and from 37.2% (ciprofloxacin) to 46.3% (amoxicillin), respectively in water. After two hours, the emerging pollutant was nearly completely removed with LMPPs, whereas MPPs removed approximately 70–75% of amoxicillin, carbamazepine, and diclofenac and 49.6% of ciprofloxacin. In wastewater samples, a percentage between 67.3% (ciprofloxacin) and 86.3% (diclofenac) was achieved in 1.5 h using LMPPs, and nearly a complete removal was obtained within 2 h. However, MPPs demonstrated a removal rate ranging from 29.8% (ciprofloxacin) to 53.2% (diclofenac) after 1.5 h treatment. In two hours, a removal range of 69.8–73.5% was attained for amoxicillin, carbamazepine, and diclofenac, and 46.3% for ciprofloxacin. Two hypothesized elimination processes could explain the superiority of LMPPs. It is expected that adsorption of pollutants onto the free sites occur first, followed by degradation of pollutants by laccases. The specific mechanisms likely involve common adsorption interactions such as covalent bonding (chemisorption), hydrogen bonding and π–π (physical adsorption)^79^. Laccase residing within the porous structure of MPPs then degrades the adsorbed pollutants. Functional groups perform an essential function in the degradation process as they get converted to free radicals, which can initiate domino reactions^80^.

**Figure 13:**
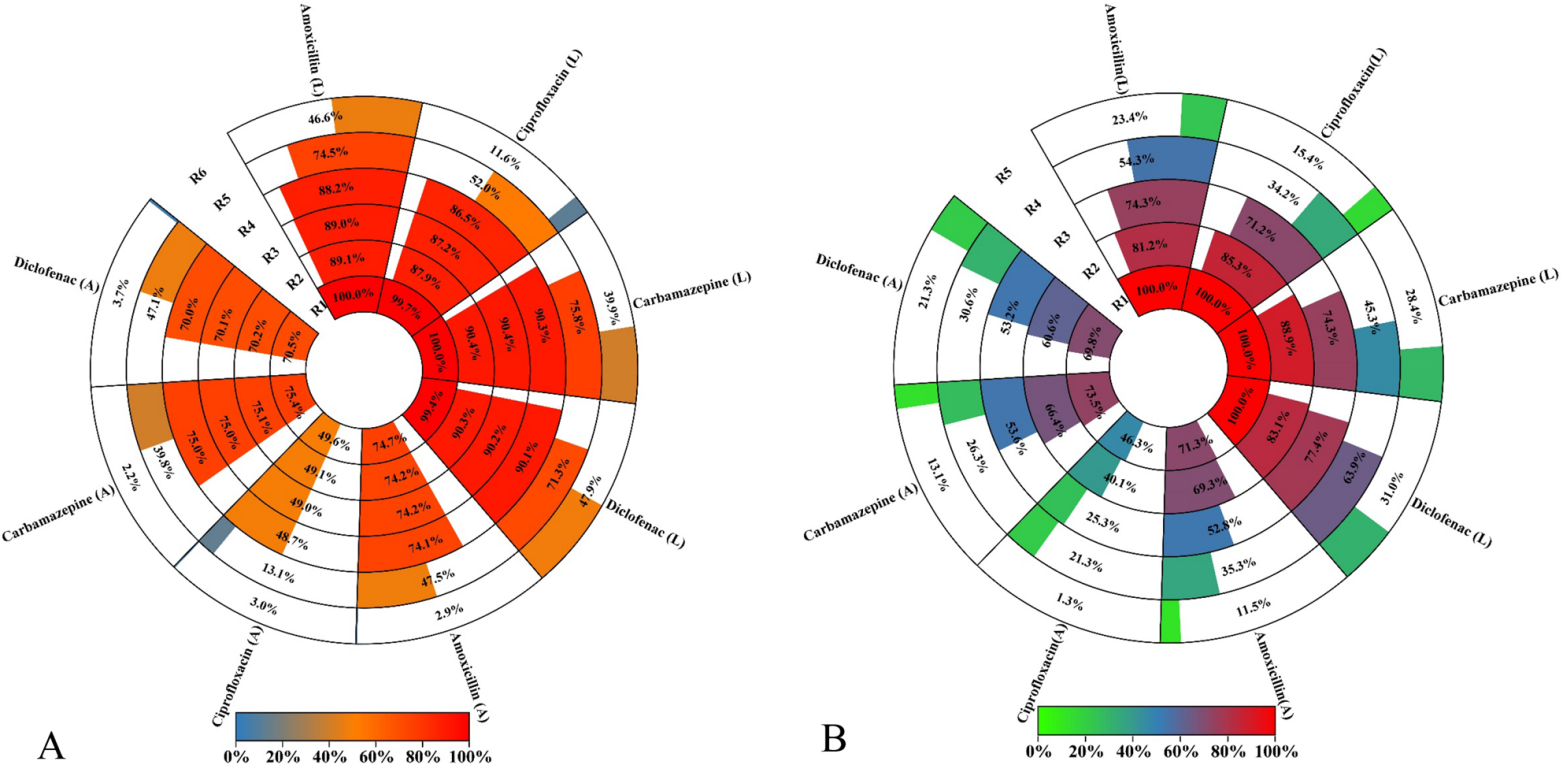
The removal efficiency of the emerging pollutants by LMPPs for water (**A**) and wastewater (**B**) over sequential cycles. The standard deviations were between 2.65 and 5.43% for MPPs and LMPPs cycles.

**Figure 14:**
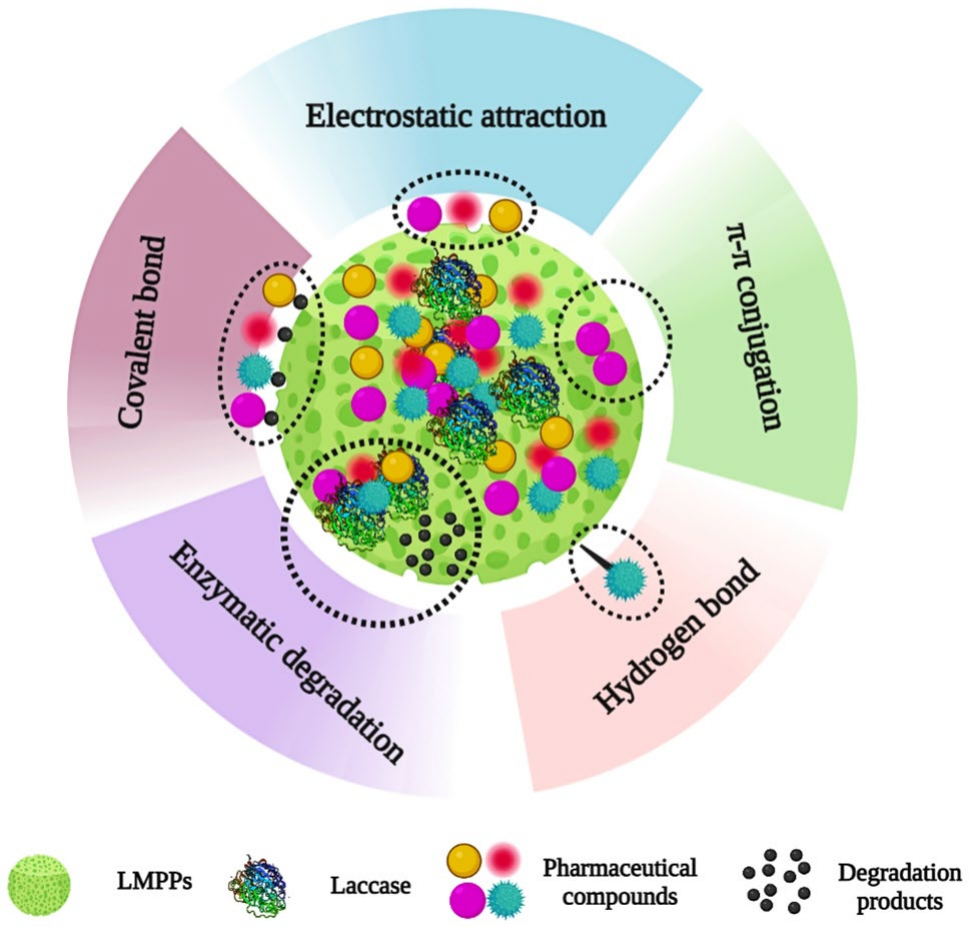
Schematic representation of degradation and removal of pharmaceuticals mechanism by LMPPs.

This system demonstrates its viability as a successful removal technique. However, its stability requires further investigation. Thus, the performance of system was gauged over six cycles of operation. Figure 13 demonstrates the system removal efficacy for six tested cycles. In all cycles, LMPPs had a better removal performance than MPPs for both water and wastewater samples. The removal of all pollutants declines as the number of cycles increases. The removal of the pollutants from wastewater lasted for five cycles while it continued up to six cycles with water. In water samples, the LMPPs can still remove the selected pollutants after six cycles with removal percentages ranging from 11.6% (ciprofloxacin) to 47.9% (amoxicillin). Consequently, using the adsorption process (MPPs) in the water medium, the system was able to perform 5 cycles with removal efficiencies ranging from 13.1% (ciprofloxacin) to 47.5% (amoxicillin). The removed pollutants by LMPPs in wastewater were better than MPPs with a removal efficiency in the 5th cycle ranging between 15.4% (ciprofloxacin) to 31% (diclofenac) for LMPPs and from 1.3% (carbamazepine) to 21.3% (diclofenac). Figure 14 depicts the proposed pharmaceutical degradation and removal using LMPPs. The decrease of catalytic effectiveness during subsequent cycles in both water and wastewater could be attributable to the occupation of adsorption sites, the scavenging of enzyme activities by other pollutants (in the case of wastewater) and the inactivation of the laccase. Additionally, the gradual reduction in the effectiveness of removal could be explained by leaching and denaturation of the laccase, as observed for ABTS oxidation^29,81^.

It can be concluded that immobilized laccase onto adsorbents can actively be degraded and simultaneously remove pharmaceuticals even from treated wastewater. Nevertheless, the economics of the entire process requires further research. Testing the capacity of this technology for the removal of a wider variety of pollutants is also essential to prove its feasibility for removing emerging pollutants.

## Conclusion

This study examined the effectiveness of activated carbon prepared from pomegranate peels as an adsorbent and laccase enzyme carrier for removing emerging pollutants such as amoxicillin, carbamazepine, ciprofloxacin, and diclofenac. The temperature of 35 °C, pH of 4, and laccase concentration of 2.5 mg/mL were determined to be the most effective immobilization parameters for achieving a 69.8% immobilization yield. Emerging pollutant adsorption onto MPPs can be characterized as an endothermic spontaneous first-order reaction following the Langmuir isotherm. LMPPs performed better than MPPs at removing pollutants in both water and wastewater samples. Long-term tests demonstrated that LMPPs was superior to MPPs in removing the target compounds from water and wastewater effluent samples. Furthermore, this study demonstrated that activated carbon derived from pomegranate peels can be utilized as a carrier for setting up adsorption-enzymatic systems for eliminating emerging pollutants while maintaining a reasonable level of stability and reusability. In addition, recycling/reusing PPs might be perceived as a low carbon innovation option and fossil free activated carbon. Nevertheless, additional research needs to be conducted to determine the technique's efficacy with a more complex sample matrix. Issues of particular interest include the competition between emerging contaminants and others for adsorption sites and their interaction with enzymes. Finally, system design parameters for a continuous large-scale process need to be investigated.

## Supplementary Information

Supplementary Information.

## Data Availability

All data generated or analysed during this study are included in this published article and its supplementary information files.
