# Supplementary material for: Efficient removal of pharmaceutical contaminants from water and wastewater using immobilized laccase on activated carbon derived from pomegranate peels
Source: Sci Rep. 2023 Jul 24;13:11933. doi: 10.1038/s41598-023-38821-3 (PMC10366155; doi:10.1038/s41598-023-38821-3)
Supplement: Supplementary file 1 — Supplementary Information. [file 41598_2023_38821_MOESM1_ESM.docx]

**Efficient removal of pharmaceutical contaminants from water and wastewater using immobilized laccase on activated carbon derived from pomegranate peels**

**Osamah J. Al-sareji ^a,b,*^, Mónika Meiczinger ^a^, Raed A. Al-Juboori ^c,d^, Ruqayah Ali Grmasha ^b,e^, Manolia Andredaki ^f^, Viola Somogyi ^a^, Ibijoke A. Idowu ^f^, Csilla Stenger-Kovács ^e,g^, Miklós Jakab ^h^, Edina Lengyel ^e,g^, Khalid S. Hashim ^f,i^**

^a^ Sustainability Solutions Research Lab, Faculty of Engineering, University of Pannonia, Egyetem str. 10, Veszprém H, 8200, Hungary

^b^ Environmental Research and Studies Center, University of Babylon, Babylon, Al-Hillah, Iraq

^c^ NYUAD Water Research Center, New York University-Abu Dhabi Campus, Abu Dhabi, P.O. Box 129188, Abu Dhabi, United Arab Emirates

^d^ Water and Environmental Engineering Research Group, Department of Built Environment, Aalto University, P.O. Box 15200, Aalto, FI-00076, Espoo, Finland

**^e^** University of Pannonia, Faculty of Engineering, Center for Natural Science, Research Group of Limnology, H-8200 Veszprem, Egyetem u. 10, Hungary

^f^ School of Civil Engineering and Built Environment, Liverpool John Moores University, UK

**^g^** ELKH-PE Limnoecology Research Group, H-8200 Veszprém, Egyetem utca 10, Hungary

^h^ Research Centre of Engineering Sciences, Department of Materials Sciences and Engineering, University of Pannonia, P.O. Box 158, H-8201 Veszprém, Hungary

^i^ Department of Environmental Engineering, College of Engineering, University of Babylon, Babylon, Al-Hillah, Iraq

* Corresponding authors: Osamah J. Al-sareji, osamah.al-sareji@unswalumni.com


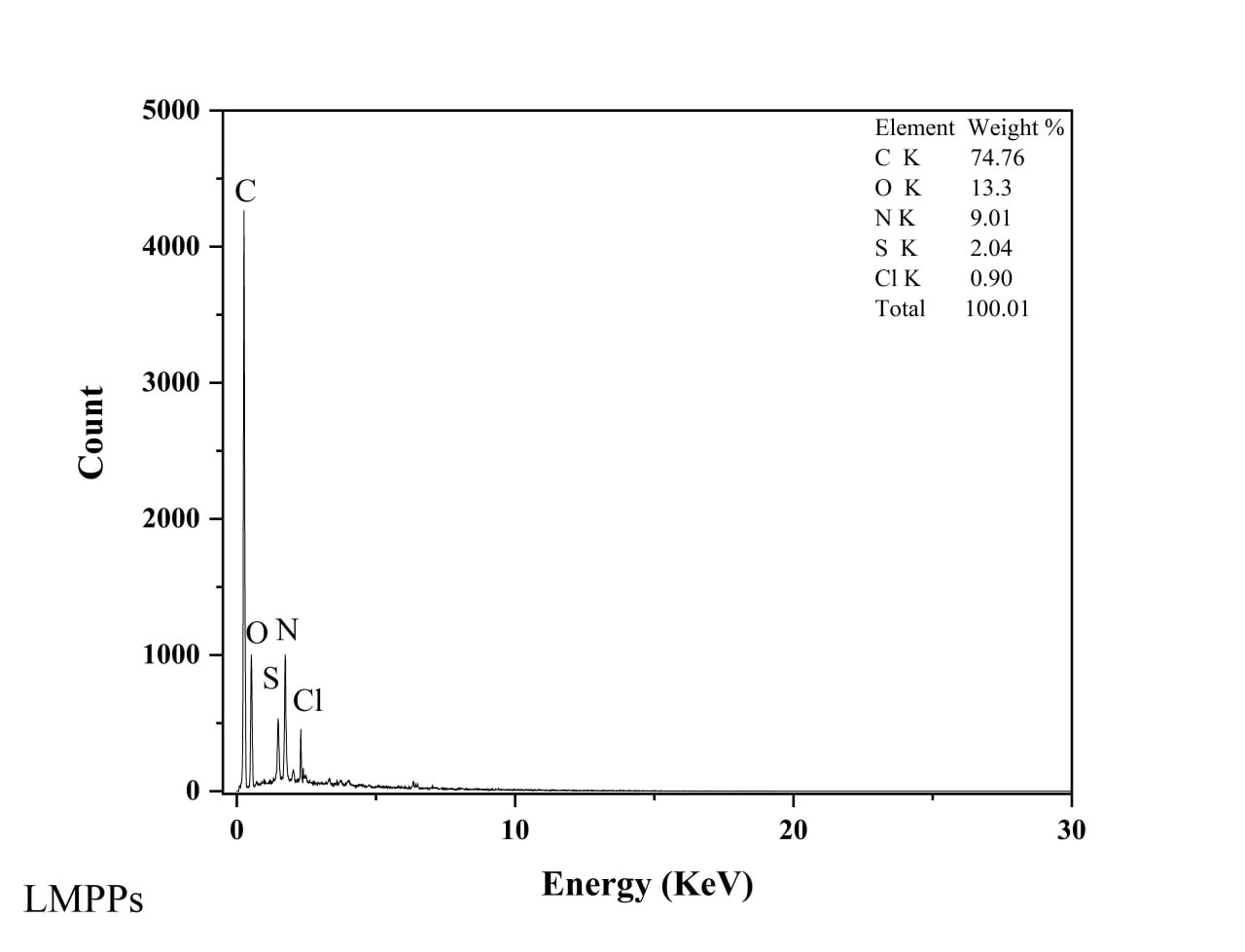


**Figure S1** EDS for MPPs and LMPPs

**Figure S2** XRD for MPPs.

| **Table S1** Residual sum of square (or error sum of square, SSE) for the isotherm and kinetic models | | | | |
| --- | --- | --- | --- | --- |
|  | Amoxicillin | Ciprofloxacin | Carbamazepine | Diclofenac |
| Langmuir | 4.7392E-6 | 1.4919E-6 | 2.5956E-6 | 1.7389E-6 |
| Freundlich | 0.0122 | 0.0054 | 0.0199 | 0.0067 |
| Pseudo-first-order | 0.0282 | 0.0145 | 0.0248 | 0.0275 |
| Pseudo-second-order | 0.0344 | 0.0239 | 0.0326 | 0.0040 |

**Table S2** Secondary efﬂuent characteristics.

| Parameters | Concentration (mg/L) |
| --- | --- |
| Chemical oxygen demand | 67.2 ±2.3 |
| Biochemical oxygen demand | 26.43 ±1.9 (5 days) |
| Suspended solids | 24.42±1.3 |
| Volatile suspended solids | 18.84± 0.8 |
| Total Phosphorus | 4.9±0.8 |
| Ammonia | 15.93±1.7 (NH_3_-NH_4_) |
| Electrical conductivity | 853±5.4 µmoss/cm |
| Total Kjeldahl nitrogen | 11.53 ± 0.7 |
| Nitrate-nitrite | 2.8 ± 0.6 (NO_2_-NO_3_) |
| Total solids | 584± 6.7 |
| Total dissolved solids | 498± 3.6 |
| pH | 6.7 |
| Temperature | 26 |
